# Gene expression results in lipopolysaccharide-stimulated monocytes depend significantly on the choice of reference genes

**DOI:** 10.1186/1471-2172-11-21

**Published:** 2010-05-04

**Authors:** Armin P Piehler, Runa M Grimholt, Reidun Øvstebø, Jens P Berg

**Affiliations:** 1 Department of Medical Biochemistry and Clinical Pharmacology, Oslo University Hospital, Ulleval, 0407 Oslo, Norway; 2 Faculty Division Ulleval University Hospital, University of Oslo, 0318 Oslo, Norway

## Abstract

**Background:**

Gene expression in lipopolysaccharide (LPS)-stimulated monocytes is mainly studied by quantitative real-time reverse transcription PCR (RT-qPCR) using GAPDH (glyceraldehyde 3-phosphate dehydrogenase) or ACTB (beta-actin) as reference gene for normalization. Expression of traditional reference genes has been shown to vary substantially under certain conditions leading to invalid results. To investigate whether traditional reference genes are stably expressed in LPS-stimulated monocytes or if RT-qPCR results are dependent on the choice of reference genes, we have assessed and evaluated gene expression stability of twelve candidate reference genes in this model system.

**Results:**

Twelve candidate reference genes were quantified by RT-qPCR in LPS-stimulated, human monocytes and evaluated using the programs geNorm, Normfinder and BestKeeper. geNorm ranked PPIB (cyclophilin B), B2M (beta-2-microglobulin) and PPIA (cyclophilin A) as the best combination for gene expression normalization in LPS-stimulated monocytes. Normfinder suggested TBP (TATA-box binding protein) and B2M as the best combination. Compared to these combinations, normalization using GAPDH alone resulted in significantly higher changes of TNF-α (tumor necrosis factor-alpha) and IL10 (interleukin 10) expression. Moreover, a significant difference in TNF-α expression between monocytes stimulated with equimolar concentrations of LPS from N. meningitides and E. coli, respectively, was identified when using the suggested combinations of reference genes for normalization, but stayed unrecognized when employing a single reference gene, ACTB or GAPDH.

**Conclusions:**

Gene expression levels in LPS-stimulated monocytes based on RT-qPCR results differ significantly when normalized to a single gene or a combination of stably expressed reference genes. Proper evaluation of reference gene stabiliy is therefore mandatory before reporting RT-qPCR results in LPS-stimulated monocytes.

## Background

Cells from the mononuclear phagocyte system play central roles in the pathophysiological processes of inflammation [1] and infection [2] Lipopolysaccharide (LPS), a cell membrane component of gram-negative bacteria, is a potent stimulator of immune responses of the mononuclear phagocyte system [3]. Stimulation of monocytes with LPS is a frequently employed model system to study inflammatory responses [4-8] and coagulation [9,10]. One approach to investigate cellular processes is by gene expression studies. Due to its high sensitivity, specificity, dynamic range and straightforwardness, quantitative reverse-transcription polymerase chain reaction (RT-qPCR) has become one of the most frequently used techniques to measure gene expression. For comparison of expression levels between certain conditions of a cell or organ gene expression quantities need to be normalized to a standard. Several approaches have been proposed to achieve adequate normalization, but expression levels of internal reference genes, habitually called housekeeping genes, are mainly used [11]. Studies from the past years have undoubtedly shown that stability assessment of internal reference genes for each experimental condition is a prerequisite for valid normalization of gene expression and reliable gene expression results [12-15]. This notion has therefore also been included in the recently published MIQE guidelines describing "*M*inimum *I*nformation for publication of *Q*uantitative real-time PCR *E*xperiments" [16].

In contrast to the fact that gene expression analysis in LPS-stimulated cells from the mononuclear phagocyte system is frequently employed - documented by about 1500 PubMed entries on "LPS, monocyte and gene expression" (as accessed by September 1^st^, 2009) - a systematic evaluation of reference gene stability has, to our knowledge, not been published yet. Moreover, a thorough review of the literature of the last years indicated that most of the published RT-qPCR results in LPS-stimulated monocytes were normalized to either GAPDH or ACTB alone, although normalization to a single reference gene is only rarely justified [16].

In the present study, mRNA expression levels of twelve candidate reference genes were assessed by RT-qPCR in human monocytes stimulated with LPS from different bacteria. The software applications geNorm [15,17], Normfinder [12,18] and Bestkeeper [14,19] were subsequently used to calculate the most stably expressed reference genes and to determine the optimal number of reference genes required for reliable normalization of gene expression data. These results were then applied to normalize expression levels of genes known to be involved in immune response and to evaluate different normalization strategies. We show that gene expression levels in LPS-stimulated monocytes are significantly dependent on the choice of reference genes which emphasizes the importance of reference gene evaluation in the frequently employed model system of LPS-stimulated monocytes

## Results

### Stability of reference gene expression

**Figure 1:**
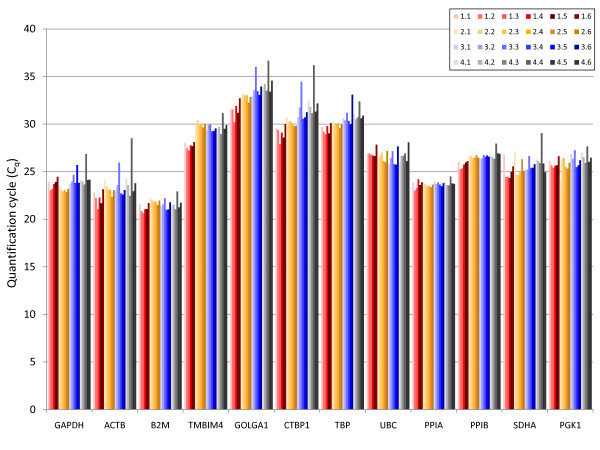
**Individual C_q _values of the candidate reference genes across all samples**. Shown are the quantification cycle (Cq) values of the candidate reference genes across all samples. 1.1-1.6 Non-stimulated monocytes at t = 0. 2.1-2.6 Non-stimulated monocytes after 3 h in culture. 3.1-3.6 Monocytes stimulated with LPS from N. meningitides for 3 h. 4.1-4.6 Monocytes stimulated with LPS from E. coli for 3 h.

To identify the most stably expressed reference genes in LPS-stimulated and non-stimulated monocytes, the expression levels of twelve candidate reference genes (Table 1) were measured by RT-qPCR in human monocytes from six healthy donors stimulated for three hours with LPS from E. coli and N. meningitides, respectively, and in non-stimulated monocytes before and after three hours in culture. Initially, descriptive statistics of expression levels were calculated by the software Bestkeeper showing that B2M and ACTB were the highest expressed genes with C_q _(quantification cycle) averages of 21.55 and 23.26, respectively (Table 2). Lowest expression was obtained with the candidate reference genes GOLGA1 and CTBP1 with mean C_q _values of 33.11 and 30.80, respectively. Figure 1 shows individual C_q _values of the investigated candidate reference genes across all samples. To determine the optimal choice and number of reference genes, the expression values of the candidate reference genes were processed in the applications geNorm [15,17], Normfinder [12,18] and Bestkeeper [14,19], which are freely available programs and generally accepted methods [16]:

**Table 1:** Candidate reference and target genes.

Candidate Reference Genes					
**Symbol**	**Name**	**Function**	**Assay ID^&^**	**r^2^**	**E**

ACTB	Actin, beta	Structural protein	Hs99999903_m1	0.997	1.97
B2M	Beta-2-microglobulin	Beta chain of MHC I	Hs99999907_m1	0.999	2.00
CTBP1	C-terminal binding protein 1	Involved in cellular proliferation.	Hs00179922_m1	0.998	2.09
GAPDH	Glyceraldehydes-3-phosphate dehydrogenase	Glycolysis and gluconeogenesis	Hs99999905_m1	0.998	2.00
GOLGA1	Golgi autoantigen, golgin subfamiliy A, 1	Unknown function	Hs00608118_m1	1.000	1.96
PGK1	Phosphoglycerate kinase 1	Glycolysis	Hs99999906_m1	0.997	2.07
PPIA	Cyclophilin A (Peptidylprolyl isomerase A)	Protein folding	Hs99999904_m1	0.996	2.08
PPIB	Cyclophilin B (Peptidylprolyl isomerase B)	Associated with the secretory pathway	Hs00168719_m1	0.996	2.00
SDHA	Succinate dehydrogenase complex, subunit A	Electron transporter	Hs00417200_m1	0.998	2.10
TMBIM4	Transmembrane BAX inhibitor motif containing 4	Involved in apoptosis	Hs00211390_m1	1.000	2.00
TBP	TATA box binding protein	Transcription factor	Hs99999910_m1	0.999	2.10
UBC	Ubiquitin C	Protein degradation	Hs00824723_m1	0.999	2.05
**Target Genes**					

**Symbol**	**Name**	**Function**	**Assay ID**	**r^2^**	Efficiency*

IL10	Interleukin 10	Antiinflammatory cytokine	Hs00174086_m1	0.989	2.01
TNF-α	Tumor necrosis factor	Proinflammatory cytokine	Hs00174128_m1	0.997	2.08

r^2^, Square of Pearson's correlation coefficient. E, Efficiency as calculated from 10-fold serial dilutions. ^&^Assay IDs from Applied Biosystems

**Table 2:** Descriptive statistics of candidate reference and target genes as calculated by BestKeeper.

Candidate Reference Genes						
	**GM [C_q_]**	**AM [C_q_]**	**Min [C_q_]**	**Max [C_q_]**	**SD [± C_q_]**	**CV [% C_q_]**

ACTB	23.26	23.30	21.05	28.52	0.92	3.95
B2M	21.55	21.55	20.62	22.92	0.40	1.87
CTBP1	30.80	30.84	27.91	36.19	1.25	4.06
GAPDH	23.89	23.91	22.87	26.86	0.58	2.43
GOLGA1	33.11	33.14	30.19	36.67	0.99	3.00
PGK1	26.08	26.09	24.08	27.66	0.57	2.17
PPIA	23.70	23.70	23.02	24.50	0.22	0.91
PPIB	26.45	26.45	25.28	27.95	0.37	1.40
SDHA	25.61	25.63	24.33	29.06	0.80	3.11
TBP	30.35	30.36	28.93	33.11	0.66	2.18
TMBIM4	29.25	29.27	27.23	31.16	0.80	2.74
UBC	26.68	26.68	25.74	28.09	0.47	1.76
**Target genes**						

	**GM [C_q_]**	**AM [C_q_]**	**Min [C_q_]**	**Max [C_q_]**	**SD [± C_q_]**	**CV [%C_q_]**

IL10	31.16	31.27	27.98	36.68	2.26	7.24
TNF-α	26.68	26.89	22.52	31.80	3.28	12.21

Descriptive statistics were calculated for all 24 samples. C_q_, quantification cycle; GM, geometric mean; AM, arithmetic mean; Min, minimum; Max, maximum; SD, standard deviation; CV, coefficient of variation.

#### i) geNorm analysis

**Figure 2:**
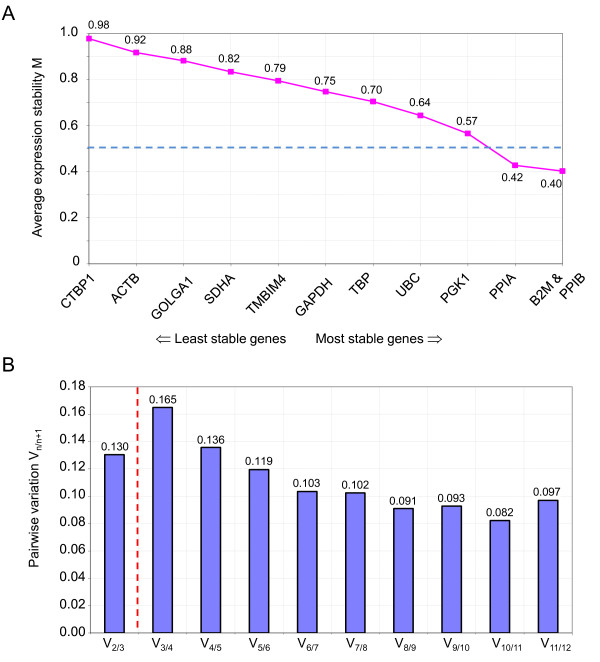
**geNorm analysis**. (A) Average expression stability M of all remaining control genes after stepwise exclusion of the least stable reference genes. More stably expressed genes are positioned on the right side of the diagram, less stably expressed on the left side. Candidate reference genes with a mean stability value M < 0.5 are regarded as stably expressed [20] as indicated by a blue, dashed line. (B) Determination of the optimal number of control genes required for reliable normalization by stepwise calculating the pariwise variation (V_n/n+1_) between normalization factors based on the n and (n+1) most stably expressed reference genes based on results results from (A). According to the developers of geNorm, a variation < 0.15 indicates no significant contribution of an additional control gene to the normalization factor and the optimal number of control genes as shown by a red, dashed line.

Expression stability of the investigated candidate reference genes in LPS-stimulated and non-stimulated monocytes as calculated by the program geNorm [15,17] are shown in Figure 1A. High gene expression variability results in high M values and indicates low expression stability. The reference genes PPIB and B2M are identified as the two most stable genes with an average expression stability M score of 0.402. As recent experimental data from the geNorm developers have shown that stably expressed genes typically exhibit mean M values lower than 0.5 in relatively homogeneous sample panels [20], the candidate reference gene PPIA can also be regarded as stably expressed with an average expression stability M value of 0.423 together with PPIB and B2M (Figure 2A). The frequently used reference genes ACTB and GAPDH performed poorly as reference genes according to the geNorm calculations and must therefore be regarded as not appropriate for gene expression normalization in monocytes stimulated with LPS.

In addition to a ranking regarding the expression stability of each gene, geNorm also calculates a normalization factor for each sample based on the most stable reference genes and reports the optimal number of required reference genes. Figure 2B illustrates the determination of the optimal number of reference genes based on the pairwise variation of sequential normalization factors with increasing number of reference genes included for normalization factor calculation. According to the geNorm developers' original publication data, the authors propose 0.15 as the cut-off, below which the optimal number of reference genes is achieved and the inclusion of an additional reference gene not required. In our study, V_2/3 _(0.13) is already below this cut-off implying that the employment of the two best-performing reference genes (PPIB and B2M) is likely to result in reliable gene expression normalization of target genes (Figure 2B). However, as the authors of the original publication explicitly recommend the minimal use of the three most stable internal control genes for normalization of RT-qPCR results [15], we decided to calculate the normalization factor based on the three best-scoring reference genes (PPIB, B2M and PPIA) for further analysis.

#### ii) Normfinder analysis

Normfinder employs a model-based approach which, in addition to the overall expression level variation, also takes intra- and intergroup variation of the candidate normalization genes into account to evaluate the expression stability [12,18]. Table 3 presents the ranking of the candidate reference genes from our study according to Normfinder. The results for intra- and intergroup variation are shown in Additional file 1. In our study, Normfinder identified ACTB as the most stably expressed reference gene with a stability value of 0.036, followed by PPIB (0.044) and GOLGA1 (0.060). For the best combination of two reference genes with a stability value of 0.024, Normfinder suggests the combination B2M and TBP with individual stability values of 0.104 and 0.100, respectively. The frequently used reference genes UBC and GAPDH were the least stable reference genes according to the Normfinder analysis, indicating that traditional reference genes exhibit substantial variation under certain circumstances.

**Table 3:** Normfinder analysis.

Gene name	Stability value
ACTB	0.036
PPIB	0.044
GOLGA1	0.060
PGK1	0.072
CTBP1	0.076
TMBIM4	0.077
PPIA	0.080
SDHA	0.091
TBP*	**0.100**
B2M*	**0.104**
GAPDH	0.118
UBC	0.168

*TBP and B2M were reported as best combination of two references genes with a stability value of 0.024

We noted that, due to different mathematical approaches of the two programs, Normfinder ranked ACTB as the most stable, single reference, whereas the same gene performed poorly in the geNorm analysis.

#### iii) Bestkeeper analysis

Bestkeeper [14,19] analyses start by calculating standard descriptive statistics for investigated genes, which are shown in Table 2 for our study. This analysis showed a high variation of the candidate reference gene CTBP1 with a standard deviation (SD) of 1.25. The developers of Bestkeeper suggest the exclusion of studied genes with SDs higher than 1.0 [14]. CTBP1 was consequently regarded as not stable and excluded from further BestKeeper analysis. Moreover, the candidate UBC exhibited with a r^2 ^of 0.046 and a p-value of 0.31 (data not shown) only a weak correlation with the geometric mean of all included candidate reference genes, which is referred to as the BestKeeper index. Hence, UBC was also excluded. All other candidate reference genes showed SDs < 1.0, with PPIA, PPIB and B2M exhibiting the lowest SDs (Table 2), which are thus regarded as the most stable candidate reference genes according to BestKeeper. All candidate reference genes were highly correlated to the BestKeeper index with coefficients of correlation between 0.646 and 0.956 and p-values < 0.001 (Table 4). Repeated pairwise correlation and regression analysis of candidate reference genes performed by BestKeeper is presented in Additional file 2.

**Table 4:** Bestkeeper correlation analysis

***BestKeeper index *vs**.	GAPDH	ACTB	B2M	TMBIM4	GOLGA1	SDHA	TBP	PGK1	PPIA	PPIB
Coefficient of correlation [r]	0.73	0.96	0.80	0.77	0.93	0.79	0.80	0.65	0.70	0.88
p-value	0.001	0.001	0.001	0.001	0.001	0.001	0.001	0.001	0.001	0.001

Unfortunately, neither the software nor the original publication presents a ranking of reference gene stability (besides SD calculations) or suggests an optimal number of reference genes for reliable normalization of expression levels based on the BestKeeper index. Taking this into account and the fact that BestKeeper is founded on similar principles as geNorm, which also identified the candidate genes PPIA, B2M and PPIB as the most stable genes, it was opted not to use BestKeeper results for further analysis.

### Evaluation of different normalization strategies

After identification of the most stably expressed reference genes in primary human monocytes stimulated with LPS, we were interested in validating our data and whether different normalization strategies significantly change gene expression results. For this, we quantified the mRNA expression of two target genes (TNF-α and IL10) known to be significantly regulated upon LPS stimulation of monocytes and, based on our data, used different approaches to normalize the expression levels of these target genes. The following normalization approaches were applied: (i) A normalization factor calculated by geNorm based on the three most stably expressed genes (PPIB, B2M and PPIA; denoted gN comb), (ii) the geometric mean of the best combination of two genes as recommended by Normfinder (B2M and TBP; denoted NF comb), (iii) the single best gene as suggested by Normfinder (ACTB), and (iv) another gene frequently used for gene expression normalization, GAPDH. Subsequently, we investigated whether these strategies resulted in significantly different expression results.

**Figure 3:**
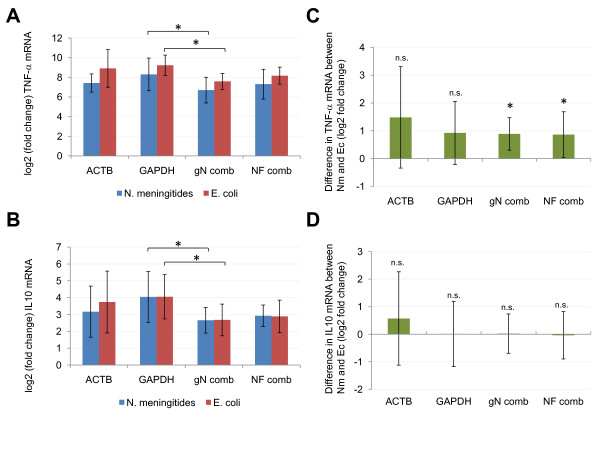
**Comparison of different normalization strategies**. Shown are mRNA expression results from different normalization strategies of TNF-α (panel A, C) and IL10 (panel B, D) in monocytes stimulated for 3 h with LPS compared to non-stimulated cells. The following normalization approaches were applied: The combinations of best-performing reference genes as calculated by geNorm (PPIB, B2M and PPIA; gN comb) or Normfinder (TBP and B2M; NF comb), the best-performing gene as indicated by Normfinder (ACTB) and another frequently used reference gene, GAPDH. (A) and (B) illustrate expression level changes as log2 of the fold change in mRNA levels between stimulated (LPS from N. meningitides, blue bars, and LPS from E. coli, red bars) and non-stimulated cells (not shown). (C) and (D) show the difference in TNF-α and IL10 mRNA, respectively, between monocytes stimulated with equimolar concentrations of LPS from N. meningitides (Nm) and E. coli (Ec) for 3 h based on different normalization approaches. Statistical significance was assessed by Wilcoxon matched pairs signed ranks test, and selected differences are indicated. Results with p < 0.05 are regarded as statistically significant and indicated by asterisks. Paired student's t test was also performed and resulted in similar findings (data not shown). n.s. not significant. Error bars indicate ± SD.

As expected, we observed an extreme increase in expression levels of TNF-α (between 104 and 596 fold change) and IL10 (between 6 and 17 fold change) in monocytes stimulated with LPS either from E. coli or N. meningitides compared to non-stimulated monocytes (Table 5, Figure 3A and 3B). These results were significant with all four normalization strategies (Table 5). However, when comparing the fold change obtained by different normalization approaches, we found that normalization with GAPDH alone resulted in a significantly higher fold change compared to the normalization strategies based on several reference genes as suggested by geNorm and Normfinder. This significant difference was observed for both TNF-α and IL10 (Figure 3A and 3B, respectively). Thus, normalization to GAPDH alone indicated significantly higher expression levels than normalization to a combination of several stably expressed genes, resulting in an overestimation of the fold change in gene expression. Gene expression levels normalized to ACTB alone showed a trend towards overestimation of expression levels compared to the normalization with a reference gene combination; however, this difference was not significant at the 0.05 level (data not shown). Another interesting finding was that we observed a significant difference in TNF-α mRNA levels in monocytes stimulated with equimolar concentrations of LPS from N. meningitides or E. coli when using a normalization strategy based on several stably expressed reference genes as suggested by geNorm or Normfinder (Figure 3C). In contrast, no significant difference between TNF-α mRNA levels in monocytes stimulated with E. coli and N. meningitides LPS, respectively, was found when normalized to either ACTB or GAPDH alone (Figure 3C). Independently of the normalization approach, no significant difference in IL10 expression was observed between monocytes stimulated with LPS from E. coli and N. meningitides, respectively (Figure 3D).

**Table 5:** Expression level changes of TNF-α and IL10 in LPS-stimulated monocytes based on different normalization strategies.

TNF-α mRNA								
	**ACTB**		**GAPDH**		**gN comb**		**NF comb**	

	**fold change**	**p-value**	**fold change**	**p-value**	**fold change**	**p-value**	**fold change**	**p-value**
Nm LPS stim/non-stim	172	< 0.05	315	< 0.05	104	< 0.05	158	< 0.05
Ec LPS stim/non-stim	480	< 0.05	596	< 0.05	192	< 0.05	288	< 0.05
								
**IL10 mRNA**								
	**ACTB**		**GAPDH**		**gN comb**		**NF comb**	

	**fold change**	**p-value**	**fold change**	**p-value**	**fold change**	**p-value**	**fold change**	**p-value**
Nm LPS stim/non-stim	9	< 0.05	16	< 0.05	6	< 0.05	8	< 0.05
Ec LPS stim/non-stim	13	< 0.05	17	< 0.05	6	< 0.05	7	< 0.05

P-values were assessed by Wilcoxon matched pairs signed ranks test. Paired Student's t test was performed as well and resulted in highly similar findings (data not shown).

gN comb, normalization based on PPIB, B2M and PPIA as suggested by geNorm; NF comb, normalization based on TBP and B2M as suggested by Normfinder; Nm, N. meningitides; Ec, E. coli; stim, stimulated.

**Figure 4:**
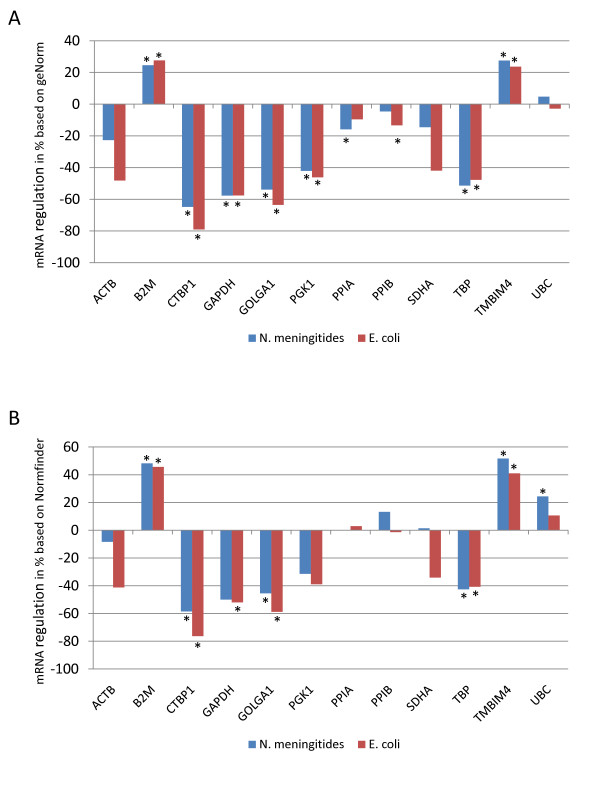
**Regulation of candidate reference genes in LPS stimulated monocytes**. Shown are mRNA level changes of candidate reference genes in LPS stimulated monocytes compared to non-stimulated cells (LPS from N. meningitides, blue bars, and LPS from E. coli, red bars). Data were normalized to the normalization factor calculated by geNorm based on the genes PPIB, B2M and PPIA (A) and to the geometric mean of the best-combination of reference genes (TBP and B2M) as suggested by Normfinder (B). Statistical significance was assessed by Wilcoxon matched pairs signed ranks test, and results with p < 0.05 are regarded as statistically significant (indicated by asterisks). Paired student's t test was also performed and resulted in similar findings (data not shown).

Differences in target gene expression results due to the choice of reference genes imply the regulation of unstable candidate reference genes during stimulation. Therefore, we calculated the mRNA level changes of the candidate reference genes between stimulated and non-stimulated cells (Figure 4). When normalizing the expression values of the candidate reference genes to the best reference gene combination as suggested by geNorm and Normfinder, respectively, we found that several candidate reference genes were significantly regulated with changes up to 80% (CTPB1) in LPS stimulated monocytes compared to non-stimulated cells (Figure 4). Here, both normalization strategies resulted in highly similar findings (Figure 4A and 4B).

## Discussion

Our study is, to our knowledge, the first report investigating expression stability of a panel of candidate reference genes in the frequently employed inflammation model of LPS-stimulated monocytes. We used three different programs (geNorm, Normfinder and Bestkeeper) to evaluate the expression stability of twelve candidate reference genes and, based on these results, investigated different normalization strategies applied to the mRNA levels of two target genes, TNF-α and IL10, in monocytes stimulated with LPS from E. coli and N. meningitides, respectively.

In LPS-stimulated monocytes, ACTB and GAPDH are mainly used as reference genes. In our study, we chose commonly used reference genes from the literature (ACTB, GAPDH, B2M, PGK1, PPIA, PPIB, SDHA, TBP and UBC) [12,14,15,21]. In addition, we included three novel reference genes, CTBP1, GOLGA1 and TMBIM4, which were identified by Lee et al. using statistical analysis of large human microarray datasets from various experimental conditions and therefore called universal housekeeping genes [22]. Published microarray-based gene expression results of resting and activated mononuclear cells would probably result in several promising candidate reference genes. Comparison of our chosen reference genes with a previous, microarray gene expression study from our group using stimulation conditions identical to this study showed that several candidate reference genes investigated here (B2M, PGK1, PPIB, TMBIM4 and UCB) were among the least regulated (fold-change <0,2) genes (data not shown) [5]. This indicates that the reference gene panel evaluated in this study included several promising candidates.

One of the main findings from our study is that several genes, like GAPDH, ACTB or UBC, which probably due to traditional reasons are still frequently used for normalization of RT-qPCR data, exhibit substantial variation in LPS-stimulated and non-stimulated monocytes. These findings clearly suggest that these genes disqualify as single internal control genes in LPS-stimulated monocytes. This observation is supported by several other studies which have shown significant variability of traditional "housekeeping" genes in different experimental conditions rendering them unsuitable as internal standards [11,13,23-29]. For example, Glare et al. demonstrated strong downregulation (10 times) of both GAPDH and ACTB in inflamed biopsy tissue of asthmatic patients compared to healthy controls or treated patients [29].

The evaluation of candidate reference gene stability depends on the method used. Surprisingly, Normfinder evaluated ACTB as the most stable single gene, whereas geNorm and BestKeeper found ACTB to be one of the least stable genes. This discrepancy is probably due to the different algorithms used in the programs. geNorm calculates the average pairwise variation for a candidate reference gene with all other tested genes and BestKeeper focuses on the coefficient of variation of a gene across all samples, whereas Normfinder employs a model-based approach which, in addition to the overall expression level variation, also takes intra- and intergroup variation into account. Even if such a discrepancy in ACTB stability has been reported before [30], the expression level changes observed in the present study resulted in highly similar findings, both for the target genes TNF-α and IL-10 (Figure 3), and for the candidate reference genes (Figure 4), when using the best combination of reference genes as suggested by geNorm or Normfinder.

Instability of normalization genes is likely to introduce a bias in reported gene expression results. In our study, we showed that normalization to GAPDH indicated significantly higher expression levels of target genes than normalization to a combination of stably expressed genes, resulting in an overestimation of the fold change in gene expression. Moreover, we observed a significant difference in TNF-α mRNA levels in monocytes stimulated with LPS from N. meningitides or E. coli when using a normalization strategy based on several stably expressed reference genes. In contrast, no significant difference between TNF-α mRNA levels in monocytes stimulated with E. coli and N. meningitides LPS, respectively, was observed when normalized to either ACTB or GAPDH alone. These false-negative results illustrate that slight changes in gene expression differences and affection of metabolic pathways may be overlooked when normalizing mRNA levels to single genes and not to a combination of stably expressed genes. Our findings that gene expression results vary depending on the chosen normalization strategy are not without precedence. For example, Dheda et al. nicely showed that a significant upregulation of IL4 in patients with tuberculosis compared to healthy controls can be masked when RT-qPCR results are normalized to an inappropriate reference gene, in this case GAPDH [13]. Moreover, treatment of tuberculosis patients led to a (nonsignificant) decrease in IL4 expression when a validated reference gene was used. In contrast, normalization of IL4 to GAPDH resulted in a 5-fold up regulation indicating persistence of the disease as opposed to the clinical, biochemical and radiological improvement of the patient [13]. Together with our results, these documentations of erroneous results introduced by the choice of inappropriate reference genes strongly advocate for the demand to make systematic validation of reference genes obligatory for reported gene expression analyses [16,31].

The absence of universal reference genes with constant expression levels in any experimental condition demands the documentation of the validity of the employed reference genes for each experimental setup. This is also true for our study, as different experimental conditions, such as type and concentration of activator, incubation time or cell type, may require a different set of valid reference genes for proper gene expression normalization. Even if the reference genes evaluated as stably expressed in the present study may be considered as candidate reference genes in subsequent studies with similar conditions of monocyte stimulation, the quest for valid reference genes must be repeated and the reference gene stability must be documented for each activation process.

## Conclusion

In the present study, we showed that gene expression levels in LPS-stimulated monocytes based on RT-qPCR results differ significantly when normalized to a single gene or a combination of stably expressed reference genes. The importance of our findings is highlighted by the fact that a review of the literature on gene expression in LPS-stimulated monocytes of the last years exhibited that the large majority of the published RT-qPCR results were normalized to a single gene, mainly GAPDH or ACTB, and that a systematic comparison of different normalization strategies was lacking. Our results indicate that normalization of RT-qPCR data to GAPDH or ACTB alone leads to imprecise gene expression results in LPS-stimulated monocytes and that the employment of several, stably expressed reference genes is mandatory. Due to the absence of universal reference genes, however, the state-of-the-art evaluation of reference gene stability has to be documented for each experimental setup and tailored to every activation process.

## Methods

### Isolation of monocytes

PBMC from six consenting, healthy donors (Blood Bank, Oslo University Hospital, Ulleval, Norway) were isolated from EDTA whole blood (450 ml) by density gradient centrifugation as previously described [32]. Monocytes were purified from PBMC by counter-current elutriation centrifugation using a Beckman J-6M/E centrifuge to a purity of >90% as assessed by flowcytometry using CD14 as marker (data not shown) and cryopreserved in RPMI 1640 (Gibco) containing 25% (v/v) fetal calf serum (FCS) and 10% (v/v) DMSO at -150°C until further use.

### Monocyte stimulation

Monocytes were thawed, resuspended and cultivated in RPMI 1640 with 5% (v/v) FCS containing 2% (v/v) of a penicillin/streptomycin solution and seeded at a density of 0.75 million cells per well in 24 wells microtiter plates (Costar). The cells were cultured at 37°C in a 5% CO_2 _atmosphere in the absence or presence of 1 μg LPS from E. coli (strain O55:B5, Cambrex BioScience) or N. meningitides (strain 44/76, B15:P1.16), respectively, in a total volume of 1 ml for 3 hours. Meningococcal endotoxin was a kindly gift of Klaus Bryn at the National Institute of Public Health, Norway [33]. After centrifugation of the microtiter plates at 500 g and 20°C for 7 minutes, the supernatants were discarded and the cells lysed in 750 μl of lysis/binding buffer (MagNa Pure LC RNA Isloation Kit-High Performance, Roche) and stored at -80°C until parallel total RNA isolation.

### RNA isolation and cDNA synthesis

Total RNA from cell lysates was isolated using the MagNa Pure LC RNA Isloation Kit-High Performance on a MagNA Pure LC Instrument (Roche) and the RNA HP blood external lysis protocol according to the manufacturer' instructions. The concentration and purity of isolated total RNA were measured using a NanoDrop ND-1000 spectrophotometer (Thermo Scientific). RNA amounts isolated from 0.75 million monocytes were between 425 ng and 995 ng. Absorbance ratios at 260/280 nm above 1.8 and a mean 260/230 nm absorbance ratio of 1.8 indicated that all RNA samples were pure and free of protein and without contamination by organic solvents. RNA integrity was assessed by micro-fluidic capillary electrophoresis using an Agilent 2100 Bioanalyzer and the RNA 6000 Pico Chip Kit (Agilent Technologies). All RNA samples exhibited RNA integrity numbers (RIN) above 9 indicating no RNA degradation and optimal RNA quality for downstream RT-qPCR applications [34].

First-strand cDNA was synthesized from 100 ng total RNA using the Omniscript RT Kit (Qiagen) and Oligo (dT) primers. The reverse transcription step was performed in duplicate at a GeneAmp PCR System 9600 (Perkin Elmer) at 37°C for 60 min and 95°C for 5 min. Finally, duplicate cDNA samples were pooled and diluted 1:2 in RNase/DNase free water prior to use in RT-qPCR. cDNAs were stored at -20°C.

### Quantitative real-time PCR (qPCR)

Traditional reference genes and genes recently proposed to be stably expressed under certain conditions were chosen from the literature [12,14,15,22,35]. cDNA specific, pre-designed primers and probes for altogether twelve candidate reference genes and two target genes were purchased from Applied Biosystems. Table 1 shows symbols and names including the assay IDs of the genes investigated in this study.

qPCR reactions were performed on a ABI 7900 HT Fast Real-Time PCR System (Applied Biosystems) in a 96-well microtiter plate format and a final volume of 20 μl using 2 μl cDNA (diluted 1:2), 10 μl 2 × TaqMan Fast Universal PCR MasterMix (Applied Biosystems), 1 μl gene specific 20 × Gene Expression Assay Mix (Applied Biosystems) and 7 μl RNase/DNase free water. The cycling conditions were as follows: 20 s polymerase activation at 95°C followed by 40 cycles of 95°C for 1s and 60°C for 20 s. All samples were amplified in duplicate and two non-template controls per primer pair were included in each run. Expression levels were obtained as individual C_q _values for each gene using the SDS RQ manager v 1.2 (Applied Biosystems) with standard settings and following instructions of the vendor. PCR efficiency for each primer-probe set was calculated using 10-fold dilutions (1:1 to 1:100 000) of pooled cDNA. Individual C_q _values were plotted against the logarithm of the dilution factor, and both Pearson's correlation coefficient (r) and PCR efficiency (E) for each assay were determined from the respective plot. r^2 ^were obtained from the lines of best fit from the Microsoft Office Excel software and PCR efficiencies were calculated according to the formula E = 10^(-1/slope) (Table 1). Efficiency plots for each gene are shown in Additional File 3.

### Analysis of gene expression data

To evaluate the gene expression stability of candidate reference genes, the software applications geNorm version 3.5 [15,17] NormFinder version 0.953 [12,18] and BestKeeper version 1 [14,19] were used according to the developers' manuals. Where necessary, raw C_q _values were transformed into linear scale expression quantities using the delta-C_q _method and E as base. Differential gene expression results were calculated using the Pfaffl method [36] when normalizing to a single gene (ACTB and GAPDH) or to the geometric mean of the best combination of two genes (B2M and TBP) as suggested by Normfinder. In the case of the Normfinder normalization strategy, the geometric mean of the B2M and TBP C_q _values in each sample were used as the reference gene C_q _in the Pfaffl equation, with E_reference gene _= 2. For normalization using the three best performing genes as suggested by geNorm (PPIB, B2M and PPIA), the according normalization factor was calculated and applied. All differential gene expression results are expressed as the geometric mean. Statistical significance was assessed by Wilcoxon matched pairs signed ranks test using SPSS version 16.0. Paired Student's t test was also performed and resulted in highly similar findings (data not shown). Results with p < 0.05 were considered statistically significant.

## Authors' contributions

APP conceived of the study, participated in the design of the study, analyzed the data, performed the statistical analysis and drafted the manuscript. RMG carried out the cell and RT-qPCR experiments, analyzed data and helped to draft the manuscript. RØ participated in the design of the study and in discussion of results. JPB participated in discussing results, coordination of the study and helped to draft the manuscript. All authors read and approved the final manuscript.

## Supplementary Material

Additional file 1 **Intra- and intergroup variation calculated by Normfinder**. The file shows the intra- and intergroup variation of each candidate reference gene as calculated by the program Normfinder. Group 1: Non-stimulated cells, group 2: LPS-stimulated cells. Click here for file

Additional file 2 **Correlation and regression analysis by BestKeeper**. The file shows the repeated pairwise correlation and regression analysis of candidate reference genes performed by BestKeeper. Click here for file

Additional file 3 **Primer efficiencies**. The file shows amplification results of ten-fold serial dilutions for each primer pair included in the study. Based on the plots, primer efficiencies and Pearson's correlation coefficient were calculated. Click here for file
